# *Baccaurea angulata* fruit juice reduces atherosclerotic lesions in diet-induced Hypercholesterolemic rabbits

**DOI:** 10.1186/s12944-017-0526-2

**Published:** 2017-07-07

**Authors:** Muhammad Ibrahim, Idris Adewale Ahmed, Maryam Abimbola Mikail, Afeez Adekunle Ishola, Samsul Draman, Muhammad Lokman Md Isa, Afzan Mat Yusof

**Affiliations:** 1 Department of Nutrition Sciences, Kulliyyah of Allied Health Sciences, International Islamic University Malaysia, 25200 Kuantan, Pahang Malaysia; 2 Department of Biotechnology, Faculty of Science, Lincoln University College, Kelana Jaya, 47301 Petaling Jaya, Selangor Malaysia; 3 Kulliyyah of Medicine, International Islamic University Malaysia, Kuantan, 25200 Malaysia; 4 Kulliyyah of Nursing, International Islamic University Malaysia, Kuantan, 25200 Malaysia; 5 Department of Biomedical Science, Kulliyyah of Allied Health Sciences, International Islamic University Malaysia, Kuantan, 25200 Malaysia

**Keywords:** Atherosclerosis, Hypercholesterolemia, Intima thickness, Lesion, Lipid profile

## Abstract

**Background:**

Atherosclerosis is the most common disease of large and medium-sized arteries linked to oxidative stress, dyslipidemia as well as chronic inflammation. The aim of this study was to evaluate the potential health benefits of *Baccaurea angulata* (BA) fruit juice on the aorta of diet-induced hypercholesterolemic rabbits, to detect an accumulation of fatty streak and evaluate the percentage of atherosclerotic lesion accrued.

**Methods:**

Thirty-five healthy male adults New Zealand White rabbits were assigned to seven different groups. Four groups were fed 1% cholesterol diet and 0, 0.5, 1.0, and 1.5 mL of BA fruit juice per kg of rabbit daily (atherogenic groups), while the other three groups were fed commercial rabbit pellet and 0, 0.5, and 1.0 mL of juice per kg of rabbit daily (normocholesterolemic groups) for 90 days. The thoracic and abdominal aorta between the heart origin and bifurcation into iliac arteries of all the rabbits were carefully removed and analyzed accordingly.

**Results:**

The supplementation of the high-cholesterol diet of hypercholesterolemic rabbits with only 0.5 mL BA/kg rabbit per day significantly (*p* < 0.001) improved aortic lipid profile, attenuated aortic fatty streak development and reduced intima thickening. Higher BA doses used (1.0 and 1.5 mL/kg rabbit per day) also significantly (*p* < 0.001) decreased further the development of aortic fatty streaks, reduced the thickening of the tunica intima layer and preserved endothelial healing following arterial injury.

**Conclusion:**

Therefore, BA fruit is a potential novel functional food with effective anti-inflammatory, anti-atherogenic and hypocholesterolemic activities.

## Background

Atherosclerosis is a complex multi-factorial disease and the major underlying pathology for cardiovascular disease (CVD), the leading cause of death worldwide. It is the most common disease of large and medium-sized arteries linked to oxidative stress, dyslipidemia as well as chronic inflammation and characterized by atherosclerotic plaque formation [1–6].

Endothelial cells, monocyte-derived macrophages, accumulation of modified lipids, expression of adhesion molecules, release of chemo-attractant chemokines, foam cells, inflamed smooth muscle cells, necrotic cores and calcified regions among others are involved in the formation of atherosclerotic lesion [7, 8]. Other immune cells as well as inflammatory cytokines and growth factors are also known to be involved in the pathogenesis of atherosclerotic processes [9]. Atherosclerosis does not simply reflect the deposition of lipids within the vessel wall of large and medium-sized arteries, it is rather a compensatory inflammatory response as a result of complex endothelial dysfunction induced by conditions such as elevated and modified low-density lipoproteins (LDL) [10], free radicals, shear stress, hypertension, infectious microorganisms, toxins or combinations of these and other factors [11].

Albeit being a substantial independent risk factor for CVD, hypercholesterolemia is shown to be manageable. The evolution of hypercholesterolemia has also been well associated with endothelial cell dysfunction characterized by decreased synthesis of nitric oxide (the most important endogenous vasodilator), local oxidation of circulating lipoproteins and their entry into the vessel wall, up-regulation of cell adhesion molecules, which facilitates adherence of monocytes to the dysfunctional endothelium, their transmigration into the sub-intimal and subsequent differentiation into macrophages, accumulation and uptake of large amounts of modified lipoproteins which results in foam cell formation, stimulation of smooth muscle cells’ migration from the media into the intima by macrophage-derived foam cells [8, 11–14].

Fruit and vegetables are highly rich in dietary fiber, vitamins, and other bioactive phytochemicals. Thus, their protective and health benefits against atherosclerosis have long been recognized [3, 5, 15, 16].

*Baccaurea angulata* (BA), an underutilized fruit, is wildly distributed in Borneo Island of Malaysia and many other parts of Indonesia. BA fruit belongs to the Euphorbiaceae family. The preliminary study on BA fruit showed that the it contains remarkable primary antioxidants which effectively scavenged free radicals and prevented lipid peroxidation in vitro and in vivo [17–23]. Therefore, the aim of the present study was to evaluate the potential health benefits BA fruit juice on the aorta of high-cholesterol fed rabbits, to detect an accumulation of fatty streak as well as to evaluate the percentage of atherosclerotic lesion accrued.

## Methods

### Materials

Healthy and fresh BA fruits were purchased from Bau, Sarawak, Malaysia. Fruit samples’ identity was also authenticated at the Forest Research Institute of Malaysia. All chemicals were from Sigma–Aldrich (Chemie, Steinheim, Germany), Merck (Darmstadt, Germany) and Nacalai-Tesque (Kyoto, Japan) while all solvents used were either analytical or chromatographic grade.

### Animal and diet

Thirty-five healthy male adult rabbits (*Oryctolagus cuniculus*; New Zealand White strain) with body weight of 2300–2800 g were obtained from A-Sapphire Enterprise, Malaysia, and were housed separately in cages in an air-conditioned room, at a temperature of 20 ± 2 °C, with a 12-h light/dark cycle and relative humidity of 50–60%. All animals were allowed free access to water and commercial rabbit pellet (Bengy, Malaysia) for 4 weeks acclimatization period before the commencement of the experiment.

The rabbits were, thereafter, randomly assigned to one of seven groups (*n* = 5). Four groups were fed 1% cholesterol diet and 0 (Positive Control), 0.5 (low dose), 1.0 (medium dose), and 1.5 mL (high dose) of BA fruit juice per kg of rabbit daily [hypercholesterolemic/atherogenic groups] [9, 24, 25], while the other three groups were fed commercial rabbit pellet and 0, 0.5, and 1.0 mL of juice per kg of rabbit daily (normocholesterolemic groups) for 90 days. Clean potable water was allowed ad libitum, and 120 g/day of food was provided during the experimental periods. All the rabbits were humanely sacrificed after the expiration of the experimental period (90 days).

The thoracic cavity was opened. The aortic root was dissected and separated from the aortic arc at the right sub-clavian branching point. The thoracic and abdominal aorta between the heart origin and bifurcation into iliac arteries were thus carefully removed and washed with ice-cold sterile physiologic phosphate buffered saline (PBS). One (1) cm section of the proximal ascending aortic arch was dissected out from each rabbit and was immediately snap-frozen in liquid nitrogen, then transferred and stored at −80 °C. Frozen aorta tissues were defrosted and homogenized in cold radio-immuno precipitation assay (RIPA) buffer containing a protease inhibitor (1 g tissue in 10 mL buffer) and then centrifuged at 8000×g for 10 min. The supernatants [10% (*w*/*v*) tissue homogenates] were collected and stored at −80 °C before analysis. Tissue homogenates were analyzed for biochemical parameters using reagent Infinity™ biochemical analyzer (Thermo Scientific, USA).

### Measurement of ox-LDL, CRP, and TBARS

Each of the oxidized-LDL (ox-LDL), C-reactive proteins (CRP), and thiobarbituric acid reactive substances (TBARS) was quantified using enzyme-linked immunosorbent assay (ELISA) kits. Both the ox-LDL and CRP assay kits were products of CUSABIO (Hubei Province, China). TBARS assay kit was purchased from Cell Biolabs, Inc. (San Diego, CA 92126, USA). The concentration of low-density lipoprotein (LDL) cholesterol was calculated using the Friedewald equation, while coronary risk indices (CRI = TC/HDL) were also estimated [26]. Admittedly, these estimations are more appropriate to human samples.

1 $$ LDL= TC\hbox{--} HDL-\left(\left[ TG\right]/2.2\right)\kern0.24em \left( all\  concentrations\  are\  in\  mmol/ L\right) $$

### Histopathological analysis

Rabbits’ thoracic aortas were carefully removed, washed with cold PBS following humane sacrifice. The thoracic aortas were immediately fixed in 4% paraformaldehyde in 0.1 M PBS (pH 7.4) for at least 48 h. Grossing was carried out to take a small portion of the organ. Five (5) mm section of the aorta was dissected from the top of the thoracic aorta. Then, they were put into tissue cassette for processing. The properly fixed aortic tissues were processed and hydrated gradually in a graded series of ethanol, using an automatic tissue processor, Leica TP 1020 (Leica Microsystem, Germany), then clarified in xylene, and embedded (Leica EG 1160, Leica Microsystem, Germany).

Tissue samples were embedded in the wax to make blocks for sectioning. Trimming and serial sectioning (3–5 sections/tissue) of tissue blocks into 5 μm thick ribbon were done by using a semi-auto rotatory microtome (Leica RM 2245, Leica Microsystem, Germany), and mounted onto super-frost Plus microscope slides (Fisher Scientific). Hematoxylin and eosin [H&E] [27], as well as van Gieson [VGS] [28] staining, were employed. The assessment and quantification of the atherosclerotic lesion accrued was carried out using previously reported procedures [29–31] with slight modification. The stained aortas were photographed, and the plaque area measured as a percentage of the lesion area using ImageJ1.47v software (National Institutes of Health, USA) with a camera (Pixlink) was quantified as:

$$ Percentage\ \left(\%\right) lesion\  area=\left( Area\  of\  the\  lesion/ Total\  aortic\  area\right)\times 100\% $$

An assessment of the structure of the aortic tissue of three rabbits from each group using transmission electron microscopy was also carried out using the reported methods [29, 32] with slight modification. Briefly, from each sample, a 1-cm section of the proximal ascending aortic arch aorta was dissected and fixed in McDowell-Trump fixative [33] prepared in 0.1 M phosphate buffer (4% formaldehyde and 1% glutaraldehyde in 0.1 M buffer), and post-fixed with 1% osmium tetroxide for 1–2 h. The properly fixed aortic tissues were dehydrated in an ethanol series, and embedded in standard epoxy (resin mixture of propylene oxide: agar low viscosity resin (1:1). After polymerization, the specimens were sectioned using ultra microtome (PT-XL Power Tome, RMC Boeckler, Inc., USA). The semi-thin sections (1 μm) were obtained after rough and fine trimming with glass knives, stained with toluidine blue stain and examined under light microscope. The blocks were further trimmed with glass and diamond knives following selection, then the ultra-thin sections (85–90 nm) were stained with lead citrate and uranyl acetate and examined under transmission electron microscope (TEM; Carl Zeiss: Libra120 Plus) at an accelerating voltage of 120 kV.

### Statistical analysis

Statistical Package for Social Sciences (SPSS, version 20.0, IBM) for Windows software was used to perform statistical analysis. The Kolmogorov-Smirnov goodness-of-fit test was also used to examine the normal distribution of all the variables. All the variables were normally distributed. The significant differences among means were compared using one-way analysis of variance (ANOVA) with Duncan’s new multiple-range post hoc test. All analyses were expressed as means ± SD (*n* = 5). Significance was determined at (*p* < 0.05 and *p* < 0.001).

## Results

### Effect of BA fruit juice on lipid profile of aorta homogenate

The effects of BA juice on the lipid profile of aorta homogenate of various rabbit groups, after 90 days of repeated administration of BA fruit juice, are shown in Table 1 [23].

**Table 1:** Effect of BA fruit Juice on lipid profile of aorta homogenate of controls and experimental rabbit groups^*^

Parameters	PC	C1	C2	C3	NgC	N1	N2
TC (mmol/L)	2.37 ± 0.06^c^	1.87 ± 0.55^c^	1.17 ± 0.38^b^	1.07 ± 0.32^b^	0.21 ± 0.04^a^	1.10 ± 0.02^b^	1.20 ± 0.03^b^
TG (mmol/L)	0.67 ± 0.21^b^	0.60 ± 0.26^b^	0.60 ± 0.20^b^	0.50 ± 0.17^b^	0.13 ± 0.06^a^	0.17 ± 0.06^a^	0.17 ± 0.12^a^
HDL (mmol/L)	0.70 ± 0.10^b^	0.87 ± 0.57^b^	0.53 ± 0.32^ab^	0.57 ± 0.25^ab^	0.10 ± 0.01^a^	0.30 ± 0.15^ab^	0.50 ± 0.36^ab^
LDL (mmol/L)	1.36 ± 0.19^d^	0.73 ± 0.17^c^	0.33 ± 0.01^b^	0.27 ± 0.05^b^	0.05 ± 0.02^a^	0.69 ± 0.16^c^	0.42 ± 0.11^b^
TC/HDL	3.43 ± 0.51^a^	2.57 ± 1.07^a^	2.43 ± 0.61^a^	1.99 ± 0.35^a^	2.10 ± 0.44^a^	3.70 ± 1.57^a^	2.56 ± 1.31^a^
Ox-LDL (μmol/mL)	4.20 ± 1.18^b^	2.44 ± 1.57^ab^	2.27 ± 1.64^ab^	1.70 ± 1.00^a^	1.55 ± 0.65^a^	1.45 ± 0.65^a^	1.40 ± 0.65^a^
CRP(pg/mL)	459.08 ± 52.68^b^	125.08 ± 41.67^a^	110.11 ± 60.96^a^	54.85 ± 6.19^a^	5.55 ± 2.99^a^	6.92 ± 2.02^a^	6.61 ± 1.99^a^
MDAE (TBARS) (μM)	44.85 ± 2.62^c^	6.42 ± 1.08^b^	4.85 ± 1.53^ab^	4.92 ± 0.81^ab^	4.80 ± 2.35^ab^	2.78 ± 0.83^a^	2.61 ± 0.33^a^

Values are means ± SD (*n* = 5). The results of all the various groups were analyzed using multivariate one-way analysis of variance (MANOVA). Values within each row not sharing a common superscript letter (a, b, c, etc.) are significantly different (*p* < 0.05) using Duncan’s new multiple-range post hoc test. PC = Positive control, C1, C2 & C3 are 0.5, 1.0 and 1.5 mL/kg/day atherogenic groups, respectively, NgC = Negative Control, while N1 and N2 are 0.5 and 1.0 mL/kg/day normal groups, respectively. Total cholesterol (TC), triacylglycerol (TG), high-density lipoprotein (HDL), low-density lipoprotein (LDL), oxidized-LDL (ox-LDL), C-reactive proteins (CRP), malondialdehyde equivalence (MDAE) and thiobarbituric acid reactive substances (TBARS)

^*^Ahmed et al. [23]

The aortic total cholesterol (TC) was significantly higher (*p* < 0.05) in the positive control group than the experimental groups of the hypercholesterolemic rabbits. The atherogenic rabbits receiving 1.0 and 1.5 mL/kg/day had a significant reduction (*p* = 0.003) in their aortic TC levels, which were 1.17 ± 0.38 and 1.07 ± 0.32 mmol/L, respectively, compared to the positive control with 2.37 ± 0.06 mmol/L aortic TC. Similar results were obtained in the aortic triacylglycerol (TG) of the hypercholesterolemic rabbits. The positive atherogenic control group had the highest aortic TG of 0.67 ± 0.21 mmol/L while the 1.5 mL/kg/day group had least TG among the atherogenic rabbits (0.50 ± 0.17 mmol/L). The aortic ox-LDL and TBARS were also higher in the positive control group than the experimental atherogenic groups. The positive and negative controls had 4.20 ± 1.18 and 1.55 ± 0.65 μmol/mL aortic ox-LDL, respectively as well as 44.85 ± 2.62 and 4.80 ± 2.3 μM aortic TBARS, respectively. The reduction in the aortic ox-LDL and TBARS of the experimental groups of hypercholesterolemic rabbits showed a dose-dependent pattern. Similarly, the aortic CRP was significantly higher (*p* < 0.000) in the positive control group than the experimental hypercholesterolemic rabbit groups. The aortic CRP levels were 459.08 ± 52.68 and 5.55 ± 2.99 pg/mL in the positive and negative controls, respectively. A distinct dose-dependent reduction pattern was also observed in the experimental groups of the hypercholesterolemic rabbits, relative to the positive control group.

### Aorta histopathology

#### Histological analysis with H&E and VGS stains

**Fig. 1:**
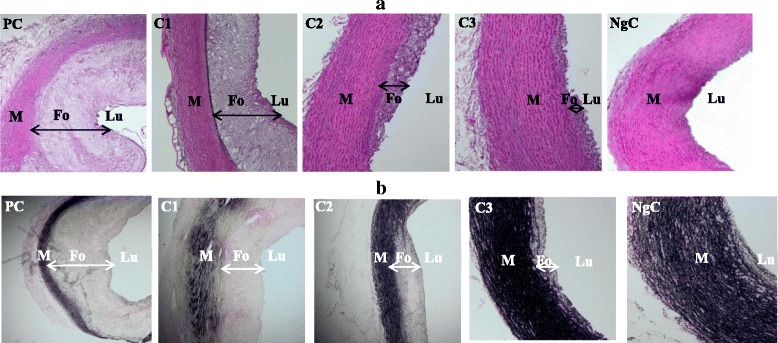
Aorta histopathology **a** H&E stain **b** van Gieson stain (Magnification ×40). Both micrograhs show a pale-staining intima thickening area representing an early athermatous lesion consisting of aggregated myointimal cells containing lipid and some fibrous intima tissues. The media, however, appears normal at this stage, because early atheroma is confined to the intima. PC = Positive Control, C1, C2 & C3 are 0.5, 1.0 and 1.5 mL/kg/day atherogenic groups, respectively, NgC = Negative Control. Lu = Lumen, Fo = Foam cells & M = Tunica media

No apparent histopathology was observed in the normocholesterolemic rabbit groups, therefore, only the negative control group was compared with the hypercholesterolemic rabbit groups. The histological examination of the aortic tissue showed apparent thickening of tunica intima, filled with foam cells, and led to a significance reduction of vascular lumens of the thoracic aorta in the positive control (PC) and low-dose (C1) groups of the hypercholesterolemic rabbits (Fig. 1a & b), whereas no apparent changes were observed in the intima surface layer of the negative control (NgC) and high dose (C3) groups. The comparison of the tunica intima and tunica media (Table 2) obtained from each rabbit (four to five aortic cross sections) showed a significant increase (*p* < 0.001) in the intima-media thickness ratio of the PC group (2.37 ± 0.06) compared to the NgC group (0.00 ± 0.00).

**Table 2:** Comparison of the tunica intima/tunica media thickness ratio and percentage atherosclerotic lesion in normo- and hypercholesterolemic rabbits

Groups	PC	C1	C2	C3	NgC
Intima: Media Thickness	2.37 ± 0.06^d^	1.60 ± 0.10^c^	0.25 ± 0.05^b^	0.10 ± 0.06^a^	0.00 ± 0.00^a^
Percentage Lesion (%)	94.37 ± 1.09^e^	53.85 ± 1.48^d^	28.13 ± 1.13^c^	11.39 ± 1.13^b^	1.60 ± 0.36^a^

Values are means ± SD (*n* = 5). The results of all the various groups were analyzed using one-way analysis of variance (ANOVA). Values within each row not sharing a common superscript letter (a, b, c, etc.) are significantly different (*p* < 0.001) using Duncan’s new multiple-range post hoc test. PC = Positive Control, C1, C2 & C3 are 0.5, 1.0 and 1.5 mL/kg/day atherogenic groups, respectively, NgC = Negative Control

**Fig. 2:**
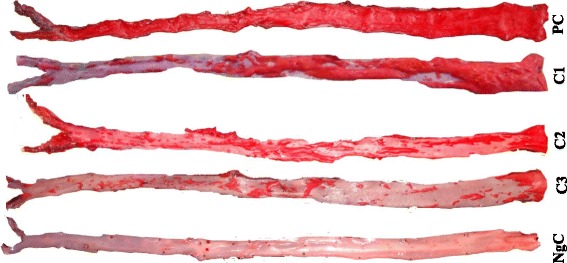
Quantification of the atherosclerotic lesions of rabbits’ aorta using ImageJ. PC = Positive Control, C1, C2 & C3 are 0.5, 1.0 and 1.5 mL/kg/day atherogenic groups, respectively, NgC = Negative Control

There was also significant (*p* < 0.001) less thickening of tunica intima: tunica media in the groups C1, C2 and C3 (1.60 ± 0.10, 0.25 ± 0.05 and 0.10 ± 0.06, respectively) compared to PC. The NgC group showed extremely little or relatively no atherosclerotic lesion (1.60 ± 0.36%). The entire aortic area was, however, significantly (*p* < 0.001) covered with atherosclerotic lesions (Table 2) in the PC group (94.37 ± 1.09%) due to cumulative effect of the chronic exposure of the aortic walls to cholesterol (Fig. 2).

The supplementation of high-cholesterol diet with BA fruit juice, however, significantly (*p* < 0.001) reduced the atherosclerotic lesions of the experimental rabbits as compared to the PC group. The various doses of BA at low, medium and high levels (C1, C2, and C3) gave percentage reduction in the atherosclerotic lesion areas of 53.85 ± 1.48, 28.13 ± 1.13 and 11.39 ± 1.13%, respectively.

#### Histological analysis under transmission electron microscopy

**Fig. 3:**
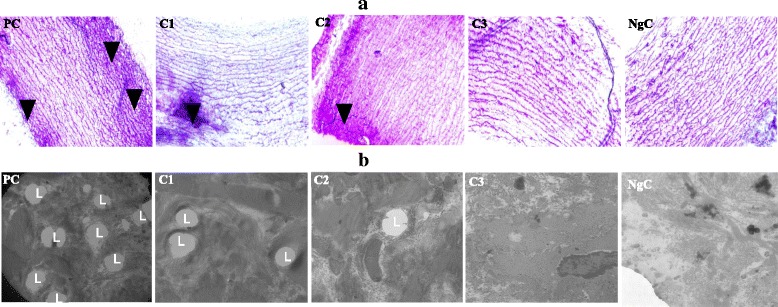
**a** semi-thin sections (1 μm) stained with toluidine blue stain examined under light microscope (Magnification ×40) & **b** ultra-thin sections (85–90 nm) stained with lead citrate and uranyl acetate examined under transmission electron microscopy. PC = Positive Control, C1, C2 & C3 are 0.5, 1.0 and 1.5 mL/kg/day atherogenic groups, respectively, NgC = Negative Control. L = Lipid-laden vacuoles

The results of the transmission electronic microscopy study showed that the experimental atherogenic groups (C1, C2 and C3) had less lipid-laden foam cells than the untreated positive control group. The result of the semi-thin sections (1 μm) stained with toluidine blue stain examined under light microscope (Fig. 3a) as well as the ultra-thin sections (85–90 nm) stained with lead citrate and uranyl acetate examined under transmission electron microscopy (Fig. 3b) showed that that BA fruit juice significantly reduced atherosclerotic lesions in the thoracic aorta of the various experimental groups treated with an increasing doses of the fruit juice, compared to the positive control group.

## Discussion

The present study has demonstrated the adverse effect of consumption of a high-cholesterol diet. It is in line with previous findings in which the consumption of a high-cholesterol diet, for a period of time, led to an increase in the oxidative state and inflammatory markers, among others [2, 9, 34].

BA fruit, however, has a potential to be used as a hypercholesterolemia management alternative to combat circulating cholesterol by preventing both its formation in the liver and its absorption from the intestine [14]. The result is in agreement with the report of the reduction of circulating triglycerides and cholesterol by green tea decoction [35] and pomegranate juice [1]. Epidemiological and animal studies have also correlated greater reduction in atherosclerosis with low LDL and high HDL levels [36–38].

The reduction in the aortic ox-LDL and TBARS of the experimental groups, compared to the positive control showed potential remarkable health benefits of the vitamins and antioxidants contained in BA fruit juice [18, 23]. Hypercholesterolemic rabbits have been reported to be susceptible to oxidative stress from an imbalance between free radical production and antioxidant level [39]. Though, the release of CRP is reported to be triggered by elevated cholesterol [14], the overproduction of several pro-inflammatory mediators is thought to be the main mechanism by which CRP induces its pro-inflammatory effects [40].

The present study, in agreement with the literature [9, 29], showed that high-cholesterol diet is responsible for the thickening of tunica intima. It also showed that the severity of the atherosclerosis lesions in the aorta is associated with hypercholesterolemia, one of the most important risk factors of endothelial dysfunction in human arteries. The significant reduction in the thickening of tunica intima of the experimental rabbit groups suggested a great anti-atherosclerotic effect of BA fruit juice. The severity of atherosclerosis lesions in cholesterol-fed rabbits have also been well evaluated using total cholesterol content of the aorta as well as plaque thickness [40].

The accumulation of cholesterol ester in foam-forming cells is responsible for the development of atheromatous lesions that usually develop in the subendothelial space. A lot of evidence has shown that oxidized LDL is responsible for cholesterol loading of macrophages foam cell formation and atherosclerosis [3]. Additionally, the important role of inflammation in the development and progression of atherosclerosis has been well studied [41]. Therefore, a significant reduction in the inflammatory lesion may be due to an anti-inflammatory action of BA fruit juice as demonstrated by the present study.

Comparatively, the concentrations of the lipid profile biomarkers were higher in the serum [23] than the aorta homogenate, with the exception of only ox-LDL and CRP. This is because the formation of foam cells, fatty streaks and fibrous plaques is as a result of the uptake of ox-LDL by the macrophage scavenger receptors (MSRs) of monocyte-derived macrophages. In addition, it has been reported that extensively oxidized LDL only occurs in the intimal area of the vessel wall, whereas, the LDLs that are found in the plasma are only mildly oxidized due to the fact that the pro-inflammatory markers such as MCP-1, cell adhesion molecules (CAMs), and selectins are functionally expressed by disturbed endothelial cells in order to recruit the circulating monocytes [42].

BA fruit could thus be assumed to possess anti-atherogenic activity, because, the supplementation of high-cholesterol diet with BA fruit juice reduced the thickening of the tunica intima layer, decreased the atherosclerotic lesion and preserved endothelial healing after the arterial injury due to hypercholesterolemia. The mechanisms thought to be involved probably include, at least in part, lowering of blood cholesterol levels, prevention of LDL oxidation as well as reduction of oxidative stress and inflammation in the injured arterial sites [2].

The present study, in consistence with the earlier studies [9, 29], showed that dietary treatment of rabbits with high-cholesterol diets caused atherosclerotic lesions in the animal model. An excess amount of cholesterol causes rapid hyperlipidemia and atherosclerosis. The reduction of the atherosclerotic lesions may be due to active compounds like vitamins, catechin and quercetin (flavonoids) as well as other polyphenols (phenolic acids and phenolic diterpenes) contained in the BA fruit matrix [18, 23], which have an anti-atherogenic effect. These bioactive compounds could have effectively worked alone or synergistically [18, 43].

The development of atherosclerosis has been associated with the oxidative modification of LDL. Therefore, the numerous and abundant antioxidant components in BA like flavonoids and other polyphenols might be responsible for the protection of LDL from oxidation. Studies have also shown that the consumption of flavonoid antioxidant is inversely related to the risk of developing coronary heart disease. Flavonoids inhibit the aggregation and adhesion of platelets in the blood, and also reduce LDL oxidation [9]. The present study of hypercholesterolemic rabbit model suggests that BA fruit may be effective as an anti-atherosclerotic agent. In this study, the supplementation of the high-cholesterol diet of the hypercholesterolemic rabbits with only 0.5 mL BA fruit juice/kg rabbit per day significantly attenuated aortic fatty streak development (*p* < 0.001). Higher BA fruit juice doses used (1.0 and 1.5 mL/kg rabbit per day) also significantly (*p* < 0.001) decreased further the development of aortic fatty streaks. One of the limitations of the present study, however, is the inability to ascertain the cause of the elevated levels of the total cholesterol and LDL-cholesterol with administration of *B. angulata* fruit juice in normocholesterolemic groups.

## Conclusion

The results of the present study demonstrated that supplementation of high-cholesterol diet in rabbits with only 0.5 mL BA fruit juice/kg rabbit per day did not only apparently improve the aortic lipid profile and attenuate aortic fatty streak development but also significantly reduced the thickening of the tunica intima layer, decreased the atherosclerotic lesion and preserved endothelial healing following the arterial injury. Higher BA fruit juice doses used (1.0 and 1.5 mL/kg rabbit per day) also significantly decreased further the development of aortic fatty streaks and improved aortic lipid profile. Therefore, the nutrient composition and antioxidant properties of BA fruit have substantially proved its potential health benefits as an effective anti-inflammatory, anti-atherogenic and hypocholesterolemic agent in the management of hypercholesterolemia-linked atheromatous lesions. Further studies are on-going to corroborate these facts.

## Abbreviations

BA: *Baccaurea angulata*
CRI: Coronary risk indices
CRP: C-reactive proteins
CVD: Cardiovascular disease
H&E: Hematoxylin and eosin
HD: High-dose
HDL: High-density lipoprotein
LD: Low-dose
LDL: Low-density lipoprotein
LDL: Low-density lipoproteins
MD: Medium-dose
MDA: Malondialdehyde
NC: Normal control
Ox-LDL: Oxidized-LDL
PC: Positive control
RIPA: Radio-immuno precipitation assay
TBARS: Thiobarbituric acid reactive substances
TC: Total cholesterol
TEM: Transmission electron microscope
TG: Total triglyceride
VGS: Van Gieson stain
